# In vitro metabolism of exemestane by hepatic cytochrome P450s: impact of nonsynonymous polymorphisms on formation of the active metabolite 17*β*‐dihydroexemestane

**DOI:** 10.1002/prp2.314

**Published:** 2017-04-27

**Authors:** Amity Peterson, Zuping Xia, Gang Chen, Philip Lazarus

**Affiliations:** ^1^ Department of Pharmaceutical SciencesWashington State UniversitySpokaneWashington

**Keywords:** Aromatase inhibitor, breast cancer, CYP450, dihydroexemestane, exemestane, pharmacogenetics, polymorphism

## Abstract

Exemestane (EXE) is an endocrine therapy commonly used by postmenopausal women with hormone‐responsive breast cancer due to its potency in inhibiting aromatase‐catalyzed estrogen synthesis. Preliminary in vitro studies sought to identify phase I EXE metabolites and hepatic cytochrome P450s (CYP450s) that participate in EXE biotransformation. Phase I metabolites were identified by incubating EXE with HEK293‐overexpressed CYP450s. CYP450s 1A2, 2C8, 2C9, 2C19, 2D6, 3A4, and 3A5 produce 17*β*‐dihydroexemestane (17*β*‐DHE), an active major metabolite, as well as two inactive metabolites. 17*β*‐DHE formation in pooled human liver microsomes subjected to isoform‐specific CYP450 inhibition was also monitored using tandem mass spectrometry. 17*β*‐DHE production in human liver microsomes was unaffected by isoform‐specific inhibition of CYP450s 2A6, 2B6, and 2E1 but decreased 12–39% following inhibition of drug‐metabolizing enzymes from CYP450 subfamilies 1A, 2C, 2D, and 3A. These results suggest that redundancy exists in the EXE metabolic pathway with multiple hepatic CYP450s catalyzing 17*β*‐DHE formation in vitro. To further expand the knowledge of phase I EXE metabolism, the impact of CYP450 genetic variation on 17*β*‐DHE formation was assessed via enzyme kinetic parameters. Affinity for EXE substrate and enzyme catalytic velocity were calculated for hepatic wild‐type CYP450s and their common nonsynonymous variants by monitoring the reduction of EXE to 17*β*‐DHE. Several functional polymorphisms in xenobiotic‐metabolizing CYP450s 1A2, 2C8, 2C9, and 2D6 resulted in deviant enzymatic activity relative to wild‐type enzyme. Thus, it is possible that functional polymorphisms in EXE‐metabolizing CYP450s contribute to inter‐individual variability in patient outcomes by mediating overall exposure to the drug and its active metabolite, 17*β*‐DHE.

**Abbreviations**
17*α*‐DHE: 17*α*‐dihydroexemestane
17*β*‐DHE: 17*β*‐dihydroexemestane
6‐HME: 6‐Hydroxymethylandrosta‐1,4,6‐triene‐3,17‐dione
AKR: aldo‐keto reductasecarbonyl reductase 1
CBR1: CYP450, cytochrome P450
EXE: exemestane
HLM: human liver microsomes
UGT2B17: uridine diphosphate glucose glucuronosyltransferase family 2 member B17 (UGT2B17)
UPLC/MS/MS: ultra‐performance liquid chromatography coupled to tandem mass spectrometry

## Introduction

EXE is a third‐generation steroidal aromatase inhibitor used in the treatment and prevention of breast cancer in postmenopausal women (Geisler et al. 1998; Goss et al. 2011). At present, a lack of consensus exists regarding the exact mechanism by which EXE irreversibly inhibits the enzyme (Brueggemeier 2002; Lombardi 2002; Ghosh et al. 2009, 2012; Viciano and Marti 2016). However, its efficacy in suppressing aromatase‐mediated synthesis of estrogens from adrenal‐supplied androgens (aromatization) is well established. Geisler et al. (1998) have previously reported mean suppression of total body aromatization of approximately 98% in postmenopausal women with advanced breast cancer treated with 25 mg EXE daily for 6–8 weeks. As the principal source of estrogen after menopause, aromatase maintains estrogen excess in hormone receptor‐positive breast cancers (Labrie 2015; To et al. 2015). Deviant estrogen signaling, in turn, promotes tumorigenesis and metastasis of hormone‐responsive mammary tumors (Sommer and Fuqua 2001; Murphy and Watson 2002; Platet et al. 2004). EXE‐induced estrogen suppression is therefore an effective treatment modality used to delay disease progression.

Although several studies have examined EXE metabolic pathways, relatively little is known about its phase I metabolites. 6‐hydroxymethylandrosta‐1,4,6‐triene‐3,17‐dione (6‐HME) has tentatively been identified as an EXE derivative in incubations with human liver microsomes (Kamdem et al. 2011). However, it is unlikely to make meaningful contributions to systemic estrogen deprivation as its potency in inhibiting aromatase is about 21‐67‐fold less potent than its parent drug (Buzzetti et al. 1993; Peterson et al. 2017). Another derivative, 17*β*‐DHE, is the major metabolite found in the plasma of individuals taking EXE. Aside from its abundance in clinical samples, 17*β*‐DHE is noteworthy for its ability to inhibit aromatase (IC_50_ = 2.3 ± 0.83 *μ*mol/L), which by one estimate is equipotent to that of EXE (IC_50_ = 1.4 ± 0.42 *μ*mol/L) (Sun et al. 2010). The concentration of 17*β*‐DHE relative to EXE in human plasma may vary fivefold between individuals although the mechanisms underlying this observation have not been fully resolved (Evans et al. 1992).

Several cytosolic reductase enzymes including carbonyl reductase 1 (CBR1) and members of the aldo‐keto reductase 1C (AKR1C) subfamily have been shown to be active in 17*β*‐DHE formation (Evans et al. 1992; Corona et al. 2009; Platt et al. 2016). Previous studies have suggested that EXE reduction may be augmented by several wild‐type CYP450s, as well (Kamdem et al. 2011). In a small panel (*n* = 15) of human liver microsomes incubated with EXE, the velocities of 17*β*‐DHE and 6‐HME formation correlated with the catalytic activity of CYP450s 1A2/4A11 and 2B6/3A, respectively (Kamdem et al. 2011).

Xenobiotic‐metabolizing members of the CYP450 family participate in the hepatic metabolism of hundreds of distinct substrates comprising approximately 75% of all marketed pharmaceuticals and catalyze many diverse reactions as reviewed by Guengerich (2001, 2008), including carbon hydroxylation, dealkylation, and epoxidation. With over 2000 known genetic variants, CYP450s are highly polymorphic enzymes (Preissner et al. 2013). A subset of variant CYP450 isoforms are associated with clinically significant alterations in drug metabolism and disease susceptibility as reviewed by Preissner et al. (2013).

Generally speaking, it is well established that nonsynonymous polymorphisms in genes involved in drug absorption, distribution, metabolism, and excretion (ADME), such as the CYP450s, can lead to genotype‐dependent variability in clinical responses for certain drugs (Kalow et al. 1998). Regarding EXE pharmacogenetics, a recent in vitro enzyme kinetics study showed that the hepatic cytosolic enzymes CBR1 and AKR1C1‐4 reduce EXE with several nonsynonymous variants in AKR1C3 and AKR1C4 significantly altering overall 17*β*‐DHE metabolism (Platt et al. 2016). While examining phase II EXE metabolism, Sun et al. (2010) found a significant correlation between uridine diphosphate glucose glucuronosyltransferase family 2 member B17 (UGT2B17) genotype in human liver microsomes (HLM) and levels of 17*β*‐DHE conjugation with glucuronic acid to form a water soluble glucuronide (glucuronidation) for urinary excretion. Overall, glucuronidation (*V*_max_/*K*_M_) of 17*β*‐DHE in the HLM panel was 36‐fold lower in UGT2B17‐null homozygotes (*2/*2) compared to wild‐type homozygotes (*1/*1) (Sun et al. 2010). These observations bolster the possibility that genetically determined differences in metabolic capacity contribute to variability in clinical responses in EXE‐treated women by influencing 17*β*‐DHE production and UGT‐driven clearance. Nonetheless, pharmacogenetic studies describing the impact of variant CYP450s on EXE metabolism are conspicuously absent from the literature. The present study expands upon the currently limited understanding of phase I EXE metabolism by identifying metabolites produced by xenobiotic‐metabolizing hepatic CYP450s. To our knowledge, this is also the first in vitro study to examine the impact of common nonsynonymous variant CYP450s on formation of the active metabolite, 17*β*‐DHE.

## Materials and Methods

### Chemicals and materials

Testosterone, boldenone, and 4‐andostene‐3,17‐dione were purchased from Hangzhou DayangChem Co., China. All other reagents used for synthesis of EXE and its phase I metabolites were ACS grade or higher and purchased from Thermo Fisher Scientific (Waltham, MA), Tokyo Chemical Industry Co. (Tokyo, Japan) or Sigma‐Aldrich (St. Louis, MO). Thin‐layer chromatography plates and silica columns used to purify the synthesized steroids were purchased from Bonna‐Agela Technologies Inc. (Wilmington, DE) and Yamazen Corp. (Osaka, Japan), respectively. Variant CYP450 overexpression vectors were made using a QuikChange II Site‐Directed Mutagenesis Kit from Agilent (Santa Clara, CA), as well as oligonucleotides manufactured by Integrated DNA Technologies (Coralville, IA). Negative control baculosomes, SuperScript II First‐Strand Synthesis System for RT‐PCR, cell culture medium, fetal bovine serum, penicillin/streptomycin, and G418 were acquired from Invitrogen (Carlsbad, CA). Choice‐Taq polymerase was purchased from Denville Scientific (Holliston, MA). LC/MS grade organic solvents, tris base, glycine, tetramethylethylenediamine (TEMED), ammonium persulfate (APS), sodium dodecyl sulfate (SDS), SuperSignal West Femto Maximum Sensitivity Substrate, Lipofectamine 2000, oligo(dT) primer, Pierce BCA protein assay kit, PVDF membranes, and monoclonal HRP‐conjugated V5 epitope antibody (catalog # MA5‐15253‐HRP) were procured from Thermo Fisher Scientific (Waltham, MA). Nonfat dry milk used as a blocking agent for Western blotting was prepared by BioRad (Hercules, CA, US). The nicotinamide adenine dinucleotide phosphate (NADPH) regeneration system included in kinetic assays was purchased from Corning (Corning, NY). Ampicillin, dithiothreitol (DTT), Tween 20, and acrylamide/bis‐acrylamide solution were acquired from Sigma‐Aldrich (St. Louis, MO). Pooled mixed gender liver microsomes from 50 human donors were obtained from XenoTech (Lenexa, Kansas, US). The ethnic composition of the pooled hepatic microsomes was 84% Caucasian, 8% Hispanic, 6% African American, and 2% Asian. (‐)‐N‐3‐benzylphenobarbital (NBP) is a product of Cypex Ltd (Dundee, United Kingdom), whereas all other compounds used for CYP450 isoform‐specific inhibition were obtained from Sigma‐Aldrich.

### Synthesis of EXE and phase I EXE metabolites

EXE and its phase I metabolites were synthesized at Washington State University using previously published protocols (Buzzetti et al. 1993; Vatèle 2007; Marcos‐Escribano et al. 2009; Varela et al. 2014; Platt et al. 2016). A Yamazen AI‐580s flash chromatography system was utilized in the purification of each compound following synthesis. Purity was estimated for each EXE derivative by photodiode array (PDA) spectrum (210–400 nm) using the Acquity I Class UPLC platform from Waters (Milford, MA). With the kind permission of Gonzaga University's Department of Chemistry (Spokane, WA), the identity of each steroid was authenticated using nuclear magnetic resonance spectra generated by a Bruker AV300 instrument (Billerica, MA). High‐resolution mass spectra were also obtained on a Waters Xevo G2‐S QTof Mass Spectrometer to confirm the parity of the experimental and theoretic mass‐to‐charge values for each compound prior to resuspension in ethanol and storage at −80°C.

### Identification of nonsynonymous polymorphisms

Common nonsynonymous polymorphisms for CYP450s 1A2, 2C8, 2C9, 2C19, 2D6, 3A4, and 3A5 were identified using the National Center for Biotechnology Information (NCBI) Variation Viewer (2015). Search filters were set to simultaneously screen dbVar and dbSNP for any missense or nonsense variants caused by single nucleotide variations, deletions, insertions, or frameshifts. Low incidence variants were excluded from the current study and defined as those occurring with a minor allele frequency (MAF) of <1% according to the GO‐ESP dataset or 1000 Genomes Project. Inter‐ethnic differences in the occurrence of common CYP450 variants were examined using NCBI's 1000 Genomes Browser. These results are summarized in Data S1.

### Creation of CYP450‐overexpressing HEK293 cell lines

Oligo(dT)_20_ was used to prime reverse transcription of pooled hepatic total RNA from five human donors (Penn State Cancer Institute Biorepository). The resultant first‐strand cDNA was further amplified using CYP450 isoform‐specific primers and *Taq* polymerase. cDNA encoding wild‐type CYP450s 1A2, 2C8, 2C9, 2C19, 2D6, 3A4, or 3A5 was introduced into the pcDNA3.1/V5‐His‐TOPO expression vector for stable overexpression in mammalian cells. CYP2D6*2 and CYP2C19*1B cDNA were also amplified from pooled human liver RNA. Additional constitutive overexpression vectors encoding nonsynonymous variants with MAF > 0.01 were produced for CYP450s 1A2, 2C8, 2C9, 2C19, and 3A4 via site‐directed mutagenesis (SDM) using wild‐type plasmid as template. Two common CYP2D6 haplotypes were likewise derived from the CYP2D6*2 overexpression vector through SDM. Oligonucleotide pairs used to amplify wild‐type CYP450 cDNA or prime mutagenesis are detailed in Data S2 and Data S3, respectively.

Each expression vector was transformed into chemically competent BL21. Transformants were then grown overnight on ampicillin selection plates at 37°C. Sanger sequencing was used for sequence confirmation prior to transfecting HEK293 with overexpression plasmids using Lipofectamine 2000 per the manufacturer instructions. Transfected HEK293 were grown for at least 3 weeks under 700 *μ*g/mL G418 selective pressure in DMEM supplemented with 4.5 g/L glucose, l‐glutamine, 110 mg/L sodium pyruvate, 10% FBS, and penicillin/streptomycin. Data S4 describes the quantification of the relative CYP450 content of each overexpressing cell line.

### Metabolite identification

CYP450‐derived phase I EXE metabolites were identified in 45‐min incubations performed at 37°C. Each 50‐*μ*L reaction contained 2.5 mmol/L EXE substrate, 3 *μ*L NADPH regeneration system, and 20 *μ*g of microsomal protein from a wild‐type CYP450‐overexpressing HEK293 cell line in phosphate‐buffered saline (PBS), pH 7.4. Similar incubations were conducted to assess background activity of endogenous metabolizing enzymes in non‐transfected HEK293, as well as commercially available negative control baculosomes. These incubations included 100 *μ*M EXE, 50 *μ*g of microsomes, or 50 *μ*g of baculosomes in addition to PBS, pH7.4, and an NADPH regeneration system. Enzymatic incubations were terminated with 50 *μ*L of ice‐cold acetonitrile before centrifugation at 13,200*g* for 15 min at 4°C. The resulting supernatant was analyzed by ultra‐pressure liquid chromatography paired with tandem mass spectrometry (UPLC/MS/MS) on a Waters ACQUITY platform configured with a 0.2 *μ*m inline filter preceding a 1.7 *μ*m ACQUITY UPLC BEH C18 column (2.1 mm × 100 mm, Ireland). The UPLC gradient used to separate EXE and its phase I metabolites has been described previously (Platt et al. 2016). 800 L/h nitrogen gas was used for drying while desolvation temperature was set at 500°C. A targeted UPLC/multiple reaction monitoring (MRM) method was performed in positive mode using electrospray ionization to monitor mass transitions for EXE (*m/z* 297.34 → 185.07), 17*α*‐DHE (*m/z* 299.14 → 135.07), 17*β*‐DHE (*m/z* 299.20 → 135.13), and 6‐HME (*m/z* 312.89 → 158.98). 0.01 sec dwell time was optimal for all four compounds, whereas 25 V of collision energy was used for EXE and 6‐HME. 20 V of collision energy was used for 17*α*‐ and 17*β*‐DHE. An additional non‐targeted screening for phase I EXE metabolites was performed using UPLC/MS to detect positively charged molecular ions with *m/z* ranging from 100 to 450. The identity of all metabolites observed was verified by comparing the observed retention times and *m/z* with those of purified standards.

### Enzyme kinetic assays

Wild‐type and variant CYP450‐mediated 17*β*‐DHE production was measured in 45‐min incubations at 37°C in PBS, pH 7.4 with varying concentrations of EXE (25–2500 *μ*mol/L). Each reaction included 20 *μ*g of microsomal protein from CYP450‐overexpressing HEK293, as well as an NADPH regeneration system. The chosen incubation length and protein concentration fell within the linear range of EXE reduction velocity curves for the seven wild‐type CYP450s assayed (data not shown). Cold acetonitrile (50 *μ*l) was used to terminate each reaction. After centrifugation for 15 min at 4°C at 13,200*g*, 17*β*‐DHE formation was monitored in the supernatant according to a previously established UPLC/MS/MS method and quantitated against a standard curve constructed of known 17*β*‐DHE concentrations (Platt et al. 2016).

### Isoform‐specific CYP450 inhibition

Reduction of EXE to 17*β*‐DHE in the presence of isoform‐specific CYP450 inhibitors was monitored in pooled human liver microsomes (HLM). Furafylline (1 *μ*mol/L) was used to inhibit CYP1A2, whereas 0.5 *μ*mol/L tranylcypromine (TCP) and 10 *μ*mol/L thioTEPA inhibited CYPs 2A6 and 2B6, respectively. Other compounds used to systematically inhibit hepatic CYP450s included 0.5 *μ*mol/L montelukast (CYP2C8 inhibitor), 1 *μ*mol/L sulfaphenazole (CYP2C9 inhibitor), 0.5 *μ*mol/L NBP (CYP2C19), 1 *μ*mol/L quinidine (CYP2D6 inhibitor), 5 *μ*mol/L clomethiazole (CYP2E1 inhibitor), and 1 *μ*mol/L ketoconazole (CYP3A inhibitor). Initial dosages were selected from the literature and then further titrated to determine the lowest dose producing maximum isoform‐specific inhibition (Bourrie et al. 1996; Administration USFaD, 2006; Khojasteh et al. 2011). Each 50‐*μ*L inhibition reaction contained 12.5 *μ*g of pooled HLM, one isoform‐specific CYP450 inhibitor dissolved in ethanol, and 10 *μ*mol/L EXE in PBS, pH 7.4. Following a 15‐min pre‐incubation at 37°C, the reactions were initiated by the addition of NADPH regeneration system and incubated for an additional 15 min before termination with 50 *μ*L of cold acetonitrile. After refrigerated centrifugation for 15 min at 13,200*g*, supernatants were collected and dried at ambient temperature in a Jouan RC10.22 vacuum concentrator. Samples were resuspended in 20 *μ*L of water/acetonitrile (1:1) and analyzed by UPLC/MS/MS using the aforementioned method. A negative control reaction was run in parallel and received ethanol vehicle rather than inhibitor. Organic solvent constituted less than 1% of each incubation.

### Statistical analyses

*K*_M_ and relative *V*_max_ values were calculated in GraphPad Prism 6 according to the Michaelis–Menten equation (GraphPad Software, Inc., San Diego, California). *V*_max_ is expressed as pmol min^−1^ mg^−1^ and was normalized to total protein content to account for differences in CYP450 expression between cell lines as determined by Western blot analysis. Two‐sided unpaired *t*‐tests were used to compare the wild‐type catalytic activity of CYP450s 1A2 and 3A4 to their respective variants. Wild‐type CYP450s 2C8, 2C9, 2C19, and 2D6 were compared to their nonsynonymous variants using one‐way ANOVA supplemented with Dunnett's multiple comparison test. In all instances, the threshold for statistical significance was set at a two‐tailed *P* < 0.05. The percent change in 17*β*‐DHE formation associated with each inhibitor in pooled HLM was likewise calculated in GraphPad Prism 6. An uninhibited reaction receiving vehicle was considered maximum catalytic activity for the purposes of comparison. All experimental results represent triplicate assays performed independently.

## Results

### Identification of phase I EXE metabolites

**Figure 1:**
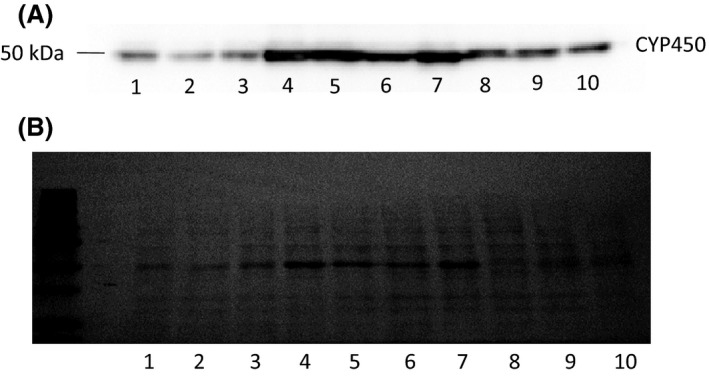
Relative quantification of overexpressed CYP450s in HEK293 microsomes. Panel (A), Detection of V5‐tagged CYP450s by Western blotting. Panel (B), Ponceau total protein staining for CYP450 normalization. Lane 1, CYP2C19; lane 2, CYP2C19^Glu92Asp^; lane 3, CYP2C19^Ile331Val^; lane 4, CYP2D6; lane 5, CYP2D6^Arg296Cys, Ser486Thr^; lane 6, CYP2D6^Pro34Ser, Ser486Thr^; lane 7, CYP2D6^Thr107Ile, Arg296Cys, Ser486Thr^; lane 8, CYP3A4; lane 9, CYP3A4^Arg162Gln^; lane 10, CYP3A5.

**Figure 2:**
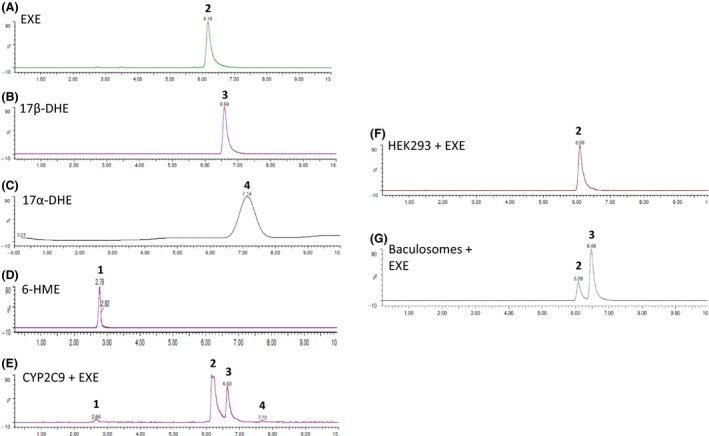
Identification of EXE metabolites. Panel (A), EXE (6‐methyleneandrosta‐1,4‐diene‐3,17‐dione) standard; panel (B), 17*β*‐DHE (17*β*‐hydroxy‐6‐methyleneandrosta‐1,4‐dien‐3‐one) standard; panel (C), 17*α*‐DHE (17*α*‐hydroxy‐6‐methyleneandrosta‐1,4‐dien‐3‐one) standard; panel (D), 6‐HME (6‐hydroxymethylandrosta‐1,4,6‐triene‐3,17‐dione) standard; panel (E), EXE metabolite profile after incubating CYP2C9‐overexpressing HEK293 microsomes with EXE; panel (F), EXE metabolite profile after incubating microsomes from the parent HEK293 cell line (no CYP450 overexpression) with EXE; panel (G), EXE metabolite profile after incubating negative control commercial baculosomes (no CYP450 overexpression) with EXE. Incubations were performed for 45 min at 37°C with 100 *μ*M exemestane and 20 *μ*g of CYP450‐overexpressing HEK293 microsomes, 50 *μ*g of non‐CYP450‐overexpressing HEK293 microsomes or 50 *μ*g of baculosomes. Peak 1, 6‐HME; peak 2, exemestane; peak 3, 17*β*‐DHE; peak 4, 17*α*‐DHE.

As shown in a representative Western blot analysis, significant levels of expression of individual CYPs were obtained in all HEK293 overexpressing cell lines (Fig. 1, panel A). Ponceau staining was used as a loading control to normalize the CYP450 content of each cell line to the amount of total protein loaded per lane (Fig. 1, panel B). The major metabolite formed in individual 45‐min incubations of EXE with CYP2C9 (Fig. 2, panel E) as well as overexpressed wild‐type CYP450s 1A2, 2C8, 2C19, 2D6, 3A4, and 3A5 (results not shown) was 17*β*‐DHE. Low levels of 6‐HME and 17*α*‐DHE formation were observed for all CYPs studied. In all cases, formation of the 17*α*‐dihydroexemestane (17*α*‐DHE) and 6‐hydroxymethylandrosta‐1,4,6‐triene‐3,17‐dione (6‐HME) metabolites exceeded the minimum threshold of detection. No EXE metabolite formation was observed in incubations using microsomes from the parent HEK293 cell line (Fig. 2, panel F), but interestingly, significant 17*β*‐DHE formation was observed using negative control baculosomes (Fig. 2, panel G). Secondary metabolite formation was not observed when overexpressing CYP450 microsomes were presented with 6‐HME or either stereoisomer of 17‐DHE as substrate (results not shown).

### Kinetic analysis of 17β‐DHE formation

**Figure 3:**
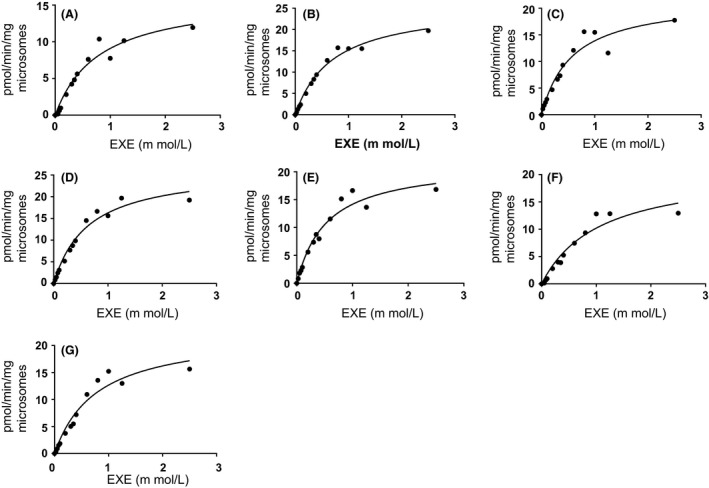
Representative kinetics curves for the reduction of exemestane to 17*β*‐DHE. Panel (A), CYP1A2; panel (B), CYP2C8; panel (C), CYP2C9; panel (D), CYP2C19; panel (E), CYP2D6; panel (F), CYP3A4; and panel (G), CYP3A5.

Representative kinetic curves of 17*β*‐DHE formation for each wild‐type hepatic CYP450 are shown in Figure 3. Kinetic assays using overexpressed CYP450 protein suggest that CYP2D6 exhibited the highest affinity for EXE (*K*_M_ = 0.57 ± 0.03 mmol/L) followed by CYP2C8 (*K*_M_ = 0.66 ± 0.10 mmol/L), CYP3A5 (*K*_M_ = 0.69 ± 0.18 mmol/L), CYP1A2 (*K*_M_ = 0.74 ± 0.5 mmol/L), and CYP3A4 (*K*_M_ = 0.83 ± 0.16 mmol/L) (Table 1). CYP450s 2C9 and 2C19 had approximately the same affinity for EXE with *K*_M_ values of 0.96 ± 0.22 mmol/L and 0.92 ± 0.18 mmol/L, respectively. CYP2C9 exhibited the slowest rate of EXE reduction, whereas CYP2C8 reduced EXE approximately fivefold faster (*V*_max_ = 26 ± 0.6 vs. 128 ± 4 pmol min^−1^ mg ^−1^). CYP1A2 catalyzed 17*β*‐DHE production at a rate of 61 ± 17 pmol min^−1^ mg ^−1^ followed by CYP2C19 (51 ± 8 pmol min^−1^ mg ^−1^) and CYP2D6 (49 ± 3 pmol min^−1^ mg ^−1^). Catalytic velocities for CYP450s 3A4 and 3A5 were not significantly different (39 ± 7 and 36 ± 4 pmol min^−1^ mg ^−1^). Intrinsic clearance (*V*_max_/*K*_M_) was calculated for each wild‐type enzyme as an indication of its overall catalytic activity against EXE. CYP2C8 exhibited the highest intrinsic clearance value (194 nL min^−1^ mg^−1^) followed by CYP2D6 (86 nL min^−1^ mg^−1^), CYP1A2 (82 nL min^−1^ mg^−1^), CYP2C19 (55 nL min^−1^ mg^−1^), CYP3A5 (52 nL min^−1^ mg^−1^), and CYP3A4 (47 nL min^−1^ mg^−1^). In addition to having the slowest rate of EXE reduction, CYP2C9 also exhibited the lowest overall catalytic activity against EXE substrate (27 nL min^−1^ mg^−1^).

**Table 1:** Kinetic analysis of wild‐type and variant CYP450s active against exemestane

Wild‐type CYP450 or variant	NCBI dbSNP identifier	Allele	*K*_M_ (mmol/L)	*V*_max_ (pmol min^−1^ mg^−1^)^a^	CL_INT_ (nl min^−1^ mg^−1^)
CYP1A2		CYP1A2*1A	0.74 ± 0.5	61 ± 17	82
CYP1A2^Ser298Arg^	rs17861157		0.59 ± 0.2^b^	80 ± 7	136
CYP2C8		CYP2C8*1A	0.66 ± 0.10	128 ± 4	194
CYP2C8^Ile269Phe^	rs11572103	CYP2C8*2	0.86 ± 0.8	280 ± 17^b^	326
CYP2C8^Arg139Lys, Lys399Arg^	rs11572080, rs10509681	CYP2C8*3	0.77 ± 0.13	144 ± 7	187
CYP2C8^Ile264Met^	rs1058930	CYP2C8*4	0.65 ± 0.3	218 ± 11^b^	335
CYP2C9		CYP2C9*1A	0.96 ± 0.22	26 ± 0.6	27
CYP2C9^Arg144Cys^	rs1799853	CYP2C9*2	0.95 ± 0.4	58 ± 4	61
CYP2C9^Ile359Leu^	rs1057910	CYP2C9*3	1.32 ± 0.16	36 ± 4	27
CYP2C9^Arg150His^	rs7900194	CYP2C9*8	1.14 ± 0.25	73 ± 13^b^	64
CYP2C9^His251Arg^	rs2256871	CYP2C9*9	1.07 ± 0.14	116 ± 13^b^	108
CYP2C19		CYP2C19*1A	0.92 ± 0.18	51 ± 8	55
CYP2C19^Ile331Val^	rs3758581	CYP2C19*1B	0.92 ± 0.13	59 ± 16	64
CYP2C19^Glu92Asp^	rs17878459		0.70 ± 0.10	59 ± 7	84
CYP2D6		CYP2D6*1A	0.57 ± 0.03	49 ± 3	86
CYP2D6^Arg296Cys, Ser486Thr^	rs16947, rs1135840	CYP2D6*2	0.95 ± 0.10^b^	23 ± 0.6^b^	24
CYP2D6^Pro34Ser, Ser486Thr^	rs1065852, rs1135840	CYP2D6*10	1.04 ± 0.01^b^	39 ± 6	38
CYP2D6^Thr107Ile, Arg296Cys, Ser486Thr^	rs28371706, rs16947, rs1135840	CYP2D6*17	0.92 ± 0.15	40 ± 2	43
CYP3A4		CYP3A4*1A	0.83 ± 0.16	39 ± 7	47
CYP3A4^Arg162Gln^	rs4986907	CYP3A4*15A	1.04 ± 0.09	46 ± 6	44
CYP3A5		CYP3A5*1A	0.69 ± 0.18	36 ± 4	52

a All *V*_max_ values were normalized to reflect the relative CYP450 content of microsomes assayed.

b Denotes that a variant exhibited statistically significant deviations (*P *<* *0.05) from the activity of its respective wild‐type CYP450.

### Impact of functional polymorphisms on EXE reduction

In all, 23 nonsynonymous polymorphisms with MAF > 0.01 were detected using the NCBI Variation Viewer to search for common variants in hepatic CYP450s involved in xenobiotic metabolism. Four polymorphisms of interest were identified for CYP2C9. Two variants were gleaned for CYP2C19, whereas a single variant was reported for both CYP450s 1A2 and 3A4. No variants matching the search criteria were listed for CYP3A5. Variation Viewer search filters returned four common single nucleotide polymorphisms (SNPs) in CYP2C8. In the present study, two of the CYP2C8 polymorphisms were investigated as a single haplotype (CYP2C8*3). In all, 11 nonsynonymous polymorphisms occur in CYP2D6 at > 1%. Although CYP2D6 is notoriously polymorphic, only a handful of common haplotypes are associated with clinically relevant alterations in drug metabolism (Zhou 2009). For this reason, only four of the SNPs identified in Variation Viewer were included in kinetic assays as the 2D6*2, 2D6*10, and 2D6*17 haplotypes.

Although *V*_max_ was comparable between wild‐type CYP1A2 and CYP1A2^Ser298Arg^, the variant enzyme exhibited significantly increased affinity for EXE with *K*_M_ values of 0.74 ± 0.5 and 0.59 ± 0.2 mmol/L, respectively. No notable differences were observed in affinity between wild‐type CYP450s 2C8, 2C9, 2C19, or 3A4 and their respective variants (Table 1). However, CYP2D6*2 (*K*_M_ = 0.95 ±0.10 mmol/L) and CYP2D6*10 (*K*_M_ = 1.04 ± 0.01 mmol/L) were both associated with decreased affinity relative to wild‐type CYP2D6 (*K*_M_ = 0.57 ± 0.03 mmol/L). The CYP2C8*2 and CYP2C8*4 polymorphisms were associated with 2.2 and 1.7‐fold increases in velocity of EXE reduction compared to wild‐type. CYP2C9*8 and CYP2C9*9 variants were likewise associated with increased rates of 17*β*‐DHE formation (*V*_max_ = 73 ± 13 and 116 ± 13 versus 26 ± 0.6 pmol min^−1^ mg^−1^ for wild‐type). The *V*_max_ for EXE reduction by CYP2D6*2 (23 ± 0.6 pmol min^−1^ mg ^−1^) is 53% lower than that of wild‐type CYP2D6 (49 ± 3 pmol min^−1^ mg ^−1^). *V*_max_ values were comparable between CYP2C19 and its variants CYP2C19^Ile331Val^ and CYP2C19^Glu92Asp^. No significant difference in *V*_max_ was observed between wild‐type CYP3A4 and CYP3A4^Arg162Gln^ (*V*_max_ = 39 ± 7 and 46 ± 6 pmol min^−1^ mg ^−1^, respectively).

### Isoform‐specific CYP450 inhibition

**Figure 4:**
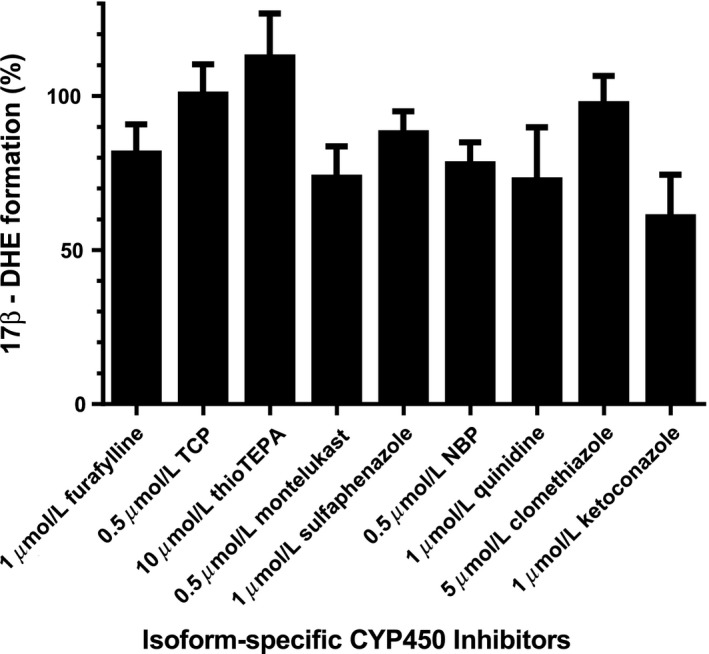
Isoform‐specific chemical inhibition of CYP450‐mediated exemestane metabolism in pooled HLM (see Materials and Methods for details). Data represent means of triplicate independent assays monitoring 17*β*‐DHE formation.

In incubations of EXE with pooled mixed gender HLMs, inhibition of CYP3A isoforms with ketoconazole resulted in a 39% decrease in 17*β*‐DHE production (Fig. 4). Isoform‐specific inhibition of CYP450s 2C8, 2C9, and 2C19 resulted in 27%, 12%, and 22% decreases in EXE reduction, respectively. Inclusion of TCP to inhibit CYP2A6 did not impact 17*β*‐DHE production to any appreciable extent (100.5 ± 5.7% control activity). Similar results were obtained when either thioTEPA or clomethiazole were included to chemically inhibit CYP2B6 (112.5 ± 8.3% control activity) or CYP2E1 (97.3 ± 5.3% control activity), respectively. Inhibition of CYP2D6 decreased EXE reduction by 27%, whereas furafylline‐induced CYP1A2 inhibition reduced formation of the active 17*β*‐DHE metabolite by 19%.

## Discussion

Previous studies examining phase I exemestane metabolism strongly suggest that reduction of EXE at the C17 carbonyl moiety to produce 17*β*‐DHE represents a major metabolic pathway for this commonly prescribed aromatase inhibitor (Evans et al. 1992; Lonning 1998; Mareck et al. 2006; Traina et al. 2008). Manufacturer‐supplied information regarding EXE metabolism is minimal and attributes metabolism in human liver preparations to CYP3A4 and members of the aldo‐keto reductase superfamily (Pfizer, 2016). CYP3A4 is further identified as the principal enzyme catalyzing EXE oxidative metabolism with subsequent formation of multiple metabolites (Pfizer, 2016). At present, a comprehensive roster of all metabolites observed in these studies has not been disclosed. A recent study has clarified the role of five cytosolic reductases in hepatic EXE metabolism in vitro (Platt et al. 2016), and multiple hepatic CYP450s may contribute to EXE metabolism as work by Kamdem et al. (2011) suggests. The present study examines the involvement of CYP450s in EXE metabolism in vitro and highlights the potential impact of common nonsynonymous CYP450 polymorphisms on 17*β*‐DHE formation.

EXE reduction to form 17*β*‐DHE was detected in incubations with HEK293‐overexpressed CYP450s 1A2, 2C8, 2C9, 2C19, 2D6, 3A4, and 3A5. These results are in agreement with previous studies indicating that EXE reduction at C17 is a major metabolic pathway and confirm that CYP3A4 catalyzes the production of multiple phase I metabolites (Evans et al. 1992; Lonning 1998; Mareck et al. 2006; Traina et al. 2008; Pfizer, 2016). Due to overlapping substrate specificity, many currently marketed pharmaceuticals are metabolized by multiple CYP450s (Preissner et al. 2013). The data presented herein bolster previous observations regarding the capacity of multiple hepatic CYP450s to engage in in vitro oxidative metabolism of EXE (Kamdem et al. 2011; Platt et al. 2016).

The relative contribution of each CYP450 to EXE metabolism in vivo is likely dependent upon differential expression in human liver, as well as their overall catalytic activity against EXE (*V*_max_/*K*_M_). CYPs 3A4 and 3A5 exhibited intermediate intrinsic clearance values in the present study (47 and 52 nL min^−1^ mg^−1^, respectively), but the literature indicates that CYP3A isoforms are well expressed in the liver comprising 30% of total CYP450 content and accounting for nearly 55% of xenobiotic metabolism (Chang and Kam 1999). The overall catalytic activity against EXE for CYP2D6 (86 nL min^−1^ mg^−1^) was second only to that of CYP2C8 (194 nL min^−1^ mg^−1^) in kinetic assays. CYP2D6 is estimated to account for 30% of drug metabolism despite constituting only 2% of total hepatic CYP450 content (Chang and Kam 1999). CYP1A2, while moderately active against EXE in vitro (CL_INT_ = 82* *nL min^−1^ mg^−1^), accounts for only 2% of overall CYP450‐mediated xenobiotic metabolism in human liver (Chang and Kam 1999). CL_INT_ values for EXE ranged from 27 to 194 nL min^−1^ mg^−1^ for CYP450 2C isoforms, which make up 20% of total hepatic CYP450 proteins but contribute only 10% of total hepatic drug metabolism (Chang and Kam 1999). Therefore, CYP3A isoforms and CYP2D6 are potentially key enzymes in phase I EXE metabolism in vivo as they are well expressed hepatically, making significant contributions to overall xenobiotic metabolism, and display high activity against EXE in vitro.

To our knowledge, only one study to date had previously examined EXE metabolism by CYP450s. In incubations with HLMs, Kamdem et al. (2011) reported two primary EXE metabolites, 17‐DHE and 6‐HME. In incubations of EXE with CYP450‐overexpressing HEK293 microsomes, we observed the formation of three EXE metabolites including 17*α*‐ and 17*β*‐DHE, as well as 6‐HME (Fig. 2). Formation of several metabolites is unsurprising as CYP450s catalyze diverse reactions (Guengerich 2001). Significantly decreased 17*β*‐DHE formation (39% reduction) in the presence of the isoform‐specific inhibitor ketoconazole suggests that CYP3As are the major hepatic CYP450s responsible for EXE reduction in human liver microsomes (Fig. 4). This result is in accordance with the manufacturer's limited description of the drug's phase I metabolism (Pfizer, 2016). Isoform‐specific inhibition of major hepatic CYP450s 1A2, 2C8, 2C9, 2C19, and 2D6 also decreased 17*β*‐DHE formation by 12–27%, which suggests that multiple CYP isoforms in addition to CYP3As may be relevant to EXE metabolism. In contrast, a previously published CYP450 inhibition experiment found that treating pooled HLM with ketoconazole strongly inhibits 6‐HME formation but had no significant effect on EXE reduction to 17*β*‐DHE (Kamdem et al. 2011). Furthermore, no effect on 17*β*‐DHE formation was observed in HLM treated with any other CYP inhibitor (Kamdem et al. 2011). The same in vitro study attributed 6‐HME formation predominantly to CYP3As and CYP2B6 (Kamdem et al. 2011). The present study cannot comment on the relative contribution of specific CYP450s to EXE oxidation to form 6‐HME. Kinetic parameters were not collected for 6‐HME formation in the present study since it exhibits low anti‐aromatase activity in vitro (Buzzetti et al. 1993; Peterson et al. 2017). In addition, while 6‐HME was a major metabolite in a previous study (Kamdem et al. 2011), it was a minor metabolite formed by the seven hepatic CYP450s tested in the present study. Discrepancies between the two studies may reflect experimental differences. It is also feasible that significant decreases in 17*β*‐DHE formation were undetectable in HLM subject to isoform‐specific inhibition in the prior study due to redundancy in EXE clearance by multiple hepatic CYP450s. Of interest, Kamdem et al. (2011) found a significant correlation between 17β‐DHE formation and the rate of activity by CYP450s 1A2 and 4A11 in a panel of HLM. CYP1A2 inhibition with furafylline decreased 17*β*‐DHE formation by approximately 19% in pooled HLM in the present study and may indeed contribute to hepatic EXE metabolism to a previously unappreciated extent.

Unfortunately, the enzyme kinetics results presented herein are not comparable to those of the previous in vitro enzyme kinetics study of EXE metabolism, which concluded that baculosome‐expressed CYP1A1, CYP2A6, and CYP4A11 are most active in 17‐DHE production (Kamdem et al. 2011). In lieu of using a commercially available baculosome system, HEK293 cell lines constitutively overexpressing CYP450s were created for kinetics assays because HEK293 are devoid of many drug‐metabolizing enzymes, including the CYP450s. In addition, significant reduction of EXE to 17*β*‐DHE was observed in negative control baculosomes (see Fig. 2). These results indicate that endogenous CYPs or active reductases are present in baculosome preparations, which is likely to confound the interpretation of kinetic assays. In contrast, background reduction in HEK293 microsomal fractions was undetectable by UPLC/MS/MS in the present study (see Fig. 2). Although overexpressed CYP2A6 was not directly tested in overexpressed HEK293 cells for activity against EXE in the present study, the involvement of CYP2A6 is questionable as it has been shown to preferentially metabolize small substrates (Zhou 2016). Furthermore, its inhibition did not appreciably decrease 17*β*‐DHE generated by HLM incubated with EXE (100.5 ± 5.7% control activity) in the present study. CYP1A1 expression, in turn, is low in human liver diminishing the likelihood that it is a key participant in phase I EXE metabolism (Stiborova et al. 2005).

Xenobiotic‐metabolizing CYP450s are highly polymorphic (Preissner et al. 2013). Multiple nonsynonymous variants often exist within a single isoform at varying levels of penetrance in the human population. CYP2D6, for instance, can harbor > 100 distinct polymorphisms several of which are associated with altered drug metabolism and increased risk for life‐threatening adverse reactions (Dalen et al. 1997; Wan et al. 2001; Gasche et al. 2004; Raimundo et al. 2004; Preissner et al. 2013). In our enzyme kinetics assays, three variant CYP450s exhibited altered affinity for EXE, whereas five deviated appreciably from their wild‐type counterparts with respect to maximum velocity of EXE reduction.

Maximum velocity of EXE reduction to form 17*β*‐DHE is similar between wild‐type CYP1A2 and CYP1A2^Ser298Arg^ (see Table 1). However, CYP1A2^Ser298Arg^ had approximately 25% higher affinity, which contributed to a 66% increase in intrinsic clearance of EXE compared to the wild‐type isoform (CL_INT = _136 vs. 82 nL min^−1^ mg^−1^). Despite its high incidence in African populations (MAF = 0.0893), a three‐dimensional structure examining conformational changes in CYP1A2 arising from arginine substitution is currently unavailable (1000 Genomes Browser, 2016; Watanabe et al. 2016). It is known, however, that amino acid residue 298 is located within a loop distal to the CYP1A2 active site (Watanabe et al. 2016). In silico analyses by Watanabe et al. (2016) predict that when the adjacent glycine at residue 299 is mutated to serine, flexibility increases near Val487 in the C‐terminal loop. Although this particular mutation does not impact overall CYP1A2 enzymatic activity as assessed by 7‐ethoxyresorufin O‐deethylation, it is indicative that polymorphisms can alter structural flexibility in loop regions (Ito et al. 2015; Watanabe et al. 2016). Structural flexibility, in turn, may strongly impact ligand‐binding induced conformational changes, substrate recognition, and overall CYP450 catalytic activity as several studies suggest (Skopalik et al. 2008; Zhang et al. 2011; Kobayashi et al. 2014; de Waal et al. 2014). Similar structural analyses are needed to determine whether CYP1A2^Ser298Arg^ exhibits deviant flexibility, possibly underlying differences observed in kinetic parameters against EXE.

CYP2C8^Ile269Phe^ (*2), CYP2C8^Arg139Lys,Lys399Arg^ (*3), and CYP2C8^Ile264Met^ (*4) are comparable to wild‐type CYP2C8 in affinity for EXE substrate in vitro. Kaspera et al. (2010) likewise found only minor differences in apparent *K*_M_ values between recombinant wild‐type CYP2C8 and its *2, *3, and *4 variants while monitoring oxidation of cerivastatin to form its O‐desmethyl‐ (M‐1) and 6‐hydroxyl‐ (M‐23) cerivastatin metabolites. Although *E*. *coli*‐expressed CYP2C8*2 yielded individual *V*_max_ values similar to wild‐type CYP2C8 for M‐1 and M‐23 formation, a 53% increase in the sum of cerivastatin metabolite clearance was noted (Kaspera et al. 2010). In the present study, CYP2C8*2 produced 17*β*‐DHE 2.2‐fold faster than wild‐type contributing to a 68% increase in overall EXE clearance. CYP2C8*2 carriers reported abdominal pain more frequently than wild‐type homozygotes in a small study of West African malaria patients taking amodiaquine, suggesting possible clinical relevance for the polymorphism (Parikh et al. 2007). The CYP2C8*3 genotype is likewise of considerable interest and has been examined extensively with regard to NSAIDS, antidiabetic agents, and 3‐hydroxy‐3‐methyl‐glutaryl‐coenzyme A (HMG‐CoA) reductase inhibitors (Kirchheiner et al. 2003; Martinez et al. 2005; Kirchheiner et al. 2006; Lopez‐Rodriguez et al. 2008; Aquilante et al. 2008; Garcia‐Martin et al. 2004). In clinical studies, CYP2C8*3 is associated with increased drug metabolism for several substrates as evidenced by significantly decreased plasma concentrations of rosiglitazone, pioglitazone, and repaglinide (Niemi et al. 2003, 2005; Kirchheiner et al. 2006; Aquilante et al. 2008; Tornio et al. 2008). In contrast, decreased ibuprofen metabolism has also been noted, implying that the kinetic properties of CYP2C8*3 may be substrate‐dependent (Garcia‐Martin et al. 2004; Martinez et al. 2005). With regard to EXE, our in vitro results indicate that the CYP2C8*3 polymorphism does not alter the drug's overall intrinsic clearance. CYP2C8*4, on the other hand, was associated with a 1.7‐fold increase in EXE clearance due to elevated *V*_max_ relative to wild‐type (218 ± 11 vs. 128 ± 4 nL min^−1^ mg^−1^). Kaspera et al. (2010) estimated that recombinant CYP2C8*4 increased the combined clearance of the M‐1 and M‐23 cerivastatin metabolites by approximately 2.5‐fold compared to wild‐type. Although CYP2C8*4 has demonstrated increased catalytic activity against both cerivastatin and EXE in vitro, human livers with the *4 genotype express lower levels of CYP2C8 protein (Kaspera et al. 2010; Naraharisetti et al. 2010). Differences in hepatic expression of variant CYPs may negate or exacerbate the overall metabolic effect of deviant catalytic activity observed in the present study. Thus, additional in vivo studies are needed to gauge what impact, if any, CYP2C8 polymorphisms have on clinical outcomes in postmenopausal breast cancer patients taking EXE.

Kinetic parameters for EXE metabolism by CYP2C9^Arg144Cys^ (*2) and CYP2C9^Ile359Leu^ (*3) were similar to those of wild‐type CYP2C9. The CYP2C9*2 and *3 polymorphisms are relatively common (MAF > 0.01) in South Asian, European, and Hispanic populations (see Data S1) and are associated with impaired warfarin metabolism (Aithal et al. 1999; Lindh et al. 2009). Although the *2 variant is associated with a poor metabolizer phenotype for certain substrates, its impact on catalytic activity is not entirely clear (Aithal et al. 1999; van der Weide et al. 2001; Rosemary et al. 2006; Lindh et al. 2009; Mosher et al. 2009). Several previous studies of the CYP2C9*2 variant are conflicting with fluvastatin and celecoxib metabolism unaffected, whereas losartan and phenytoin clearance significantly decreased relative to wild type (Aynacioglu et al. 1999; Sandberg et al. 2002; Yasar et al. 2002; Kirchheiner et al. 2003). Impaired warfarin metabolism is likewise associated with CYP2C9*3 necessitating the need for genotype‐based dose reductions in clinical settings (Aithal et al. 1999; Lindh et al. 2009). Work by Wei et al. (2007) suggests that CYP2C9*2 and *3 exhibit altered metabolism due to decreased coupling efficiency in the P450 catalytic cycle. Another study posits that the substrate binding pocket of CYP2C9*3 is enlarged relative to wild type, resulting in reduced enzymatic activity against warfarin (Sano et al. 2010). CYP2C9^Arg150His^ (*8) and CYP2C9^His251Arg^ (*9) polymorphic protein reduced EXE to 17*β*‐DHE 2.8‐ and 4.5‐fold faster than wild‐type CYP2C9, respectively. The CYP2C9*8 allele also increased clearance of the antidiabetic tolbutamide in vitro (Blaisdell et al. 2004). In vivo, the CYP2C9*8 allele is associated with decreased phenytoin metabolism due to strong linkage disequilibrium with SNPs in the gene promoter that downregulates the expression (Allabi et al. 2005; Cavallari et al. 2013). In silico analyses by Matimba et al. (2009) predicted reduced activity of CYP2C9*9 compared to wild‐type. However, a significant correlation between the CYP2C9*9 allele and phenytoin metabolism was not detected in African epilepsy patients (Matimba et al. 2009). Published discrepancies in variant CYP2C9 activity are common and likely arise from variations in experimental procedures between laboratories (Jarrar and Lee 2014).

CYP2D6 is believed to be the most polymorphic of the major drug‐metabolizing hepatic CYP450s (Preissner et al. 2013). A 72% decrease in EXE clearance was observed for CYP2D6^Arg296Cys, Ser486Thr^ (*2) relative to wild‐type CYP2D6 due to significant decreases in affinity and 17*β*‐DHE formation rate. Sakuyama et al. (2008) reported a similar two‐fold decrease in substrate affinity in transiently expressed CYP2D6*2 while monitoring bufuralol 1′‐hydroxylation. Although analysis of the crystal structure of CYP2D6 suggests that residue 296 may be involved in substrate recognition, a study of CYP2D6*2A in Europeans found no association with altered drug metabolism (Marez et al. 1997; Rowland et al. 2006). In the present study, HEK293‐expressed CYP2D6^Pro34Ser, Ser486Thr^ (*10) increased the *K*_M_ value for EXE substrate by 82% causing a 56% decrease in clearance compared to CYP2D6*1. CYP2D6*10 is highly prevalent in Asians and has previously been associated with decreased catalytic activity in vitro (Johansson et al. 1994; Bradford 2002; Ji et al. 2002; Ishiguro et al. 2004; Shen et al. 2007; Sakuyama et al. 2008). Shen et al. (2007) estimated that CYP2D6*10 protein decreases catalytic activity in a substrate‐dependent manner with intrinsic clearance of probe substrates reduced to 4–28% that of wild‐type protein. Diminished catalytic activity by the *10 variant is attributed to increased enzyme instability as a result of serine substitution in a proline‐rich region near the N‐terminus (Yokota et al. 1993; Sakuyama et al. 2008). CYP2D6^Thr107Ile, Arg296Cys, Ser486Thr^ (*17) is common in individuals of African heritage and is likewise considered a reduced function allele (Aklillu et al. 1996; Masimirembwa et al. 1996; Griese et al. 1999; Wennerholm et al. 1999; Zhou 2009). CYP2D6*17 exhibits considerable variability in catalytic activity against various substrates (Zhou 2009). Studies using recombinant CYP2D6*17 protein have reported increased metabolism of certain substrates, such as haloperidol, but decreased metabolism of others, including codeine (Oscarson et al. 1997; Bogni et al. 2005). The Thr107Ile substitution (rs28371706) is believed to alter a highly conserved region of CYP2D6 involved in substrate recognition, perhaps explaining the substrate‐dependent effects previously reported (Gotoh 1992; Koymans et al. 1993; Hasler et al. 1994). It is interesting to note that recombinant CYP2D7*17 protein yielded a 61% decrease in EXE affinity relative to wild type although this observation did not reach statistical significance.

Kinetic parameters measuring substrate affinity (*K*_M_) and production of the major active metabolite 17*β*‐DHE (*V*_max_) indicate that EXE metabolism by common allelic variants of CYP2C19 and CYP3A4 is comparable to that of their respective wild‐type CYP450s. The neutral effect of the CYP2C19^Ile331Val^ (*1B) polymorphism on EXE reduction confirms prior observations of equivalent catalytic activity for the CYP2C19*1A and *1B alleles (Blaisdell et al. 2002). Although the CYP2C19^Glu92Asp^ SNP (rs17878459) did not directly impact 17*β*‐DHE formation, it cosegregates with approximately 20% of CYP2C19*2 poor metabolizing alleles in Caucasians (Ibeanu et al. 1998). Found predominantly in Africans, the CYP3A4^Arg162Gln^ (*15A) polymorphism does not appreciably impact EXE metabolism. However, kinetic studies of the variant enzyme with additional substrates are needed to confirm its overall effect on CYP3A4 activity.

The present in vitro study augments existing pharmaceutical knowledge by examining the role of hepatic CYP450s in the metabolism of EXE, a widely used endocrine therapy for hormone‐responsive breast cancer. Qualitative enzymatic incubations with EXE confirm that multiple hepatic monoxygenases from CYP450 families 1, 2, and 3 catalyze the production of 6‐HME, 17*α*‐DHE, as well as the active metabolite 17*β*‐DHE. Earlier studies suggested that 17*β*‐DHE may partially determine overall drug exposure by acting as an androgen agonist, contributing to estrogen blockade through aromatase inhibition, and by serving as a gateway to phase II conjugation and excretion (Ariazi et al. 2007; Sun et al. 2010). Thus, any genetic factors influencing 17*β*‐DHE formation or clearance may contribute to inter‐individual variation in the overall therapeutic efficacy of EXE by altering a major metabolic pathway. This possibility is bolstered by the observation that three of the variant CYP450s included in this study had altered affinity for EXE substrate leading to differential 17*β*‐DHE production while five variants had deviant catalytic rates of EXE reduction. To our knowledge, this is the first study to report the impact of common nonsynonymous polymorphisms in CYP450s on EXE reduction to its 17*β*‐DHE metabolite. A previous in vitro study demonstrated the capacity of genetic variation to alter EXE metabolism by the cytosolic ketosteroid reductases CBR1 and AKR1C1‐4 (Platt et al. 2016). Although differences in experimental procedures preclude a direct comparison of kinetic parameters between the two studies, it appears that both cytosolic and microsomal phase I enzymes contribute to in vitro EXE metabolism. An important limitation of the overexpression model used in this study was the inability to assess the metabolic impact of copy number variations or polymorphisms in noncoding promoter regions. Additional studies are needed to determine whether multiple hepatic CYP450s contribute to overall EXE metabolism in vivo and whether common genetic variants in phase I enzymes are associated with differential metabolite production or clinical outcomes in EXE‐treated breast cancer patients.

## Disclosure

There are no conflicts of interest.

## Supporting information


**Data S1.** Digital Content 1.doc.

**Data S2.** Digital Content 2.doc.

**Data S3.** Digital Content 3.doc.

**Data S4.** Digital Content 4.doc.
