# Supplementary material for: In vitro metabolism of exemestane by hepatic cytochrome P450s: impact of nonsynonymous polymorphisms on formation of the active metabolite 17β‐dihydroexemestane
Source: Pharmacol Res Perspect. 2017 Apr 27;5(3):e00314. doi: 10.1002/prp2.314 (PMC5464343; doi:10.1002/prp2.314)
Supplement: Supplementary file 1 — Data S1. Digital Content 1.doc. [file PRP2-5-e00314-s001.doc]

**Supplemental 1.** Inter-ethnic differences in the minor allele frequency of common nonsynonymous polymorphisms in CYP450s.

| Polymorphism | NCBI dbSNP  Identifier | 1000G  MAF | GO-ESP  MAF | South Asian (SAS)  MAF | European (EUR)  MAF | African (AFR)  MAF | Hispanic  (AMR)  MAF | East Asian (EAS)  MAF |
| --- | --- | --- | --- | --- | --- | --- | --- | --- |
| CYP1A2^Ser298Arg^ | rs17861157 | 0.0240 | 0.0239 | 0 | 0 | 0.0893 | 0.0029 | 0 |
| CYP2C8^Ile269Phe^ | rs11572103 | 0.0547 | 0.0554 | 0.0123 | 0.004 | 0.1891 | 0.0115 | 0 |
| CYP2C8^Arg139Lys^ | rs11572080 | 0.0457 | 0.0853 | 0.0297 | 0.1183 | 0.0083 | 0.0994 | 0.001 |
| CYP2C8^Lys399Arg^ | rs10509681 | 0.0457 | 0.0854 | 0.0297 | 0.1183 | 0.0083 | 0.0994 | 0.001 |
| CYP2C8^Ile264Met^ | rs1058930 | 0.0166 | 0.0406 | 0.0072 | 0.0577 | 0.0083 | 0.0187 | 0 |
| CYP2C9^Arg144Cys^ | rs1799853 | 0.0479 | 0.0955 | 0.0348 | 0.1243 | 0.0083 | 0.0994 | 0.001 |
| CYP2C9^Ile359Leu^ | rs1057910 | 0.0485 | 0.0484 | 0.1094 | 0.0726 | 0.0023 | 0.0375 | 0.0337 |
| CYP2C9^Arg150His^ | rs7900194 | 0.0148 | 0.0201 | 0.001 | 0.002 | 0.053 | 0.0014 | 0 |
| CYP2C9^His251Arg^ | rs2256871 | 0.0220 | 0.0268 | 0 | 0.001 | 0.0817 | 0.0014 | 0 |
| CYP2C19^Ile331Val^ | rs3758581 | 0.0485 | 0.0473 | 0.1094 | 0.0686 | 0.0023 | 0.0346 | 0.0397 |
| CYP2C19^Glu92Asp^ | rs17878459 | 0.0090 | 0.0231 | 0 | 0.0358 | 0.0038 | 0.0058 | 0 |
| CYP2D6^Arg296Cys^ | rs16947 | 0.3592 | 0.4004 | 0.3620 | 0.3429 | 0.5537 | 0.3271 | 0.1399 |
| CYP2D6^Ser486Thr^ | rs1135840 | 0.4012 | 0.4083 | 0.4724 | 0.4543 | 0.3238 | 0.5245 | 0.2956 |
| CYP2D6^Pro34Ser^ | rs1065852 | 0.2380 | 0.1885 | 0.1646 | 0.2018 | 0.1127 | 0.1484 | 0.5714 |
| CYP2D6^Thr107Ile^ | rs28371706 | 0.0591 | 0.0585 | 0 | 0.002 | 0.2179 | 0.0086 | 0 |
| CYP3A4^Arg162Gln^ | rs4986907 | 0.0052 | 0.0103 | 0 | 0.001 | 0.0174 | 0.0029 | 0 |
