# Supplementary material for: In vitro metabolism of exemestane by hepatic cytochrome P450s: impact of nonsynonymous polymorphisms on formation of the active metabolite 17β‐dihydroexemestane
Source: Pharmacol Res Perspect. 2017 Apr 27;5(3):e00314. doi: 10.1002/prp2.314 (PMC5464343; doi:10.1002/prp2.314)
Supplement: Supplementary file 2 — Data S2. Digital Content 2.doc. [file PRP2-5-e00314-s002.doc]

| CYP450 | 5' → 3' Oligonucleotide Sequence |
| --- | --- |
| CYP1A2 | tacagatggcattgtccca |
|  | gttgatggagaagcgcag |
| CYP2C8 | acaatggaaccttttgtggtcc |
|  | gacagggatgaagcagatctgg |
| CYP2C9 | gagaaggcttcaatggattc |
|  | gacaggaatgaagcacag |
| CYP2C19 | acaatggatccttttgtggtcc |
|  | gacaggaatgaagcacagctgat |
| CYP2D6 | ttggtagtgaggcaggtatgg |
|  | gcggggcacagcacaaa |
| CYP3A4 | agtagtgatggctctcatcccag |
|  | ggctccacttacggtgc |
| CYP3A5 | gaagaaggaaagtggcgatgg |
|  | ttctccacttagggttccatctct |
