# Supplementary material for: In vitro metabolism of exemestane by hepatic cytochrome P450s: impact of nonsynonymous polymorphisms on formation of the active metabolite 17β‐dihydroexemestane
Source: Pharmacol Res Perspect. 2017 Apr 27;5(3):e00314. doi: 10.1002/prp2.314 (PMC5464343; doi:10.1002/prp2.314)
Supplement: Supplementary file 3 — Data S3. Digital Content 3.doc. [file PRP2-5-e00314-s003.doc]

| Nonsynonymous CYP450 | 5' → 3' Oligonucleotide Sequence for Site-Directed Mutagenesis |
| --- | --- |
| CYP1A2 Ser298Arg | ggatgaggttgcctctggctctaggcc |
|  | ggcctagagccagaggcaacctcatcc |
| CYP2C8 Ile264Met | tgatcaggaagcaatccataaagtcccgaggattg |
|  | caatcctcgggactttatggattgcttcctgatca |
| CYP2C8 Ile269Phe | cttttcctgctccattttgaacaggaagcaatcgataaagt |
|  | actttatcgattgcttcctgttcaaaatggagcaggaaaag |
| CYP2C8 Arg139Lys  Lys399Arg | cggtcctcaatgctcttcttccccatcccaa |
|  | ttgggatggggaagaagagcattgaggaccg  agatatttggattaggaaattccttgtcatcatgtagcacggaag  cttccgtgctacatgatgacaaggaatttcctaatccaaatatct |
| CYP2C9 Arg144Cys | gcttcctcttgaacacagtcctcaatgctcctc |
|  | gaggagcattgaggactgtgttcaagaggaagc |
| CYP2C9 Arg150His | cctccacaaggcagtgggcttcctcttga |
|  | tcaagaggaagcccactgccttgtggagg |
| CYP2C9 His251Arg | gttgttcatgtccattgattcttggcgttcttttactttttccaaaatata |
|  | tatattttggaaaaagtaaaagaacgccaagaatcaatggacatgaacaac |
| CYP2C9 Ile359Leu | gtggggagaaggtcaaggtatctctggacctcg |
|  | cgaggtccagagataccttgaccttctccccac |
| CYP2C19 Glu92Asp | cctcttccagaaaactcgtctccaagatcaatcag |
|  | ctgattgatcttggagacgagttttctggaagagg |
| CYP2D6 Pro34Thr  Ser486Thr | gggggcctggtgtgtagcgtgcagc |
|  | gctgcacgctacacaccaggccccc  gtcagccaccactatgcgcaggttctcatcattga  tcaatgatgagaacctgcgcatagtggtggctgac |
| CYP2D6 Thr107Ile  Arg296Cys  Ser486Thr | caggatctggatgatgggcacaggcggg |
|  | cccgcctgtgcccatcatccagatcctg |
| CYP3A4 Arg162Gln | gcctgtctctgcttcctgcctcagatttctcac |
|  | gtgagaaatctgaggcaggaagcagagacaggc |
