# Supplementary material for: In vitro metabolism of exemestane by hepatic cytochrome P450s: impact of nonsynonymous polymorphisms on formation of the active metabolite 17β‐dihydroexemestane
Source: Pharmacol Res Perspect. 2017 Apr 27;5(3):e00314. doi: 10.1002/prp2.314 (PMC5464343; doi:10.1002/prp2.314)
Supplement: Supplementary file 4 — Data S4. Digital Content 4.doc. [file PRP2-5-e00314-s004.docx]

**Supplemental 4**. CYP450 quantification in HEK293 cell lines.

Following antibiotic selection, the relative CYP450 content of each overexpressing cell line was assessed via Western blotting. Briefly, CYP450-overexpressing HEK293 cells were resuspended in PBS 1:1 followed by four flash freeze-thaw cycles. The homogenate was centrifuged at 9,000 g for 30 min at 4˚C. The supernatant was then subjected to 1 h centrifugation at 34,000 g in a chilled Beckman L7-65 ultracentrifuge. To remove cytosolic contamination, the supernatant was discarded before resuspending the microsomal fraction in 1.5 ml PBS. After an additional 60-min refrigerated centrifugation at 34,000 g, the supernatant was again discarded. The washed pellet was resuspended in PBS and stored at -80˚C. The bicinchoninic acid assay (BCA) was used to determine protein concentration prior to SDS-PAGE. 7.5 μg of microsomal protein from each wildtype or variant cell line was loaded onto a 10% tris-glycine polyacrylamide gel and run at 125 volts for approximately 1 h. Gel-embedded proteins were then transferred onto PVDF (0.45 μm pore size) for 90 minutes using 30 V. After a 1-h incubation in 5% milk in tris-buffered saline with 0.1% Tween (TBST) at room temperature, the membrane was incubated with HRP-conjugated anti-V5 antibody (1:7500) overnight at 4˚C. Following a 30-min wash in TBST to remove excess antibody, V5-tagged recombinant CYP450 proteins were visualized on a ChemiDoc Imager using SuperSignal West Femto Maximum Sensitivity Substrate. Ponceau staining served as a loading control. Band density was measured using Image J (NIH, Bethesda, MD, US).
